# Night-to-night variability in respiratory outcomes from home sleep testing in children with suspected sleep-disordered breathing: a retrospective analysis using real-world data

**DOI:** 10.1007/s11325-026-03677-0

**Published:** 2026-05-16

**Authors:** Samuel Tschopp, Arina Reist, Urs Borner, Marco Caversaccio, Philipp Latzin, Insa Korten

**Affiliations:** 1 Department of Otorhinolaryngology - Head and Neck Surgery, Bern University Hospital, Inselspital, University of Bern, Bern, Switzerland; 2 Division of Paediatric Respiratory Medicine and Allergology, Department of Paediatrics, Inselspital, Bern University Hospital, University of Bern, Bern, Switzerland; 3 Department of Otorhinolaryngology - Head and Neck Surgery, Inselspital, Bern University Hospital and University of Bern, Freiburgstrasse 20, Bern, 3010 Switzerland

**Keywords:** obstructive sleep apnea, sleep-disordered breathing, pediatric, diagnosis, night-to-night variability, home sleep apnea testing

## Abstract

**Purpose:**

Pediatric obstructive sleep-disordered breathing (SDB) is frequently diagnosed using single-night home sleep apnea testing (HSAT). However, concerns persist regarding diagnostic accuracy due to night-to-night variability and the first-night effect, defined as systematic differences between the first and subsequent nights. Previous research on pediatric polysomnography has yielded conflicting findings regarding these phenomena. This study assesses the presence of a first-night effect and quantifies night-to-night variability in respiratory parameters among children undergoing HSAT.

**Methods:**

This retrospective study analyzed respiratory polygraphy data collected over two consecutive nights from a real-world, unselected population of children with suspected SDB. The primary outcome was the difference in apnea-hypopnea index (AHI) between the first and second nights, commonly referred to as the first-night effect. Secondary outcomes included differences in other respiratory parameters and between-night accuracy in diagnosing obstructive sleep apnea (OSA).

**Results:**

Forty-eight children (median age: 6.7 years, 75% male) were included in the analysis. No systematic differences were detected between the first and second nights in AHI or other respiratory parameters, suggesting an absence of the first-night effect. Nevertheless, for some participants, measurements varied widely between nights. This resulted in 29% of children changing the diagnostic category of OSA between nights.

**Conclusion:**

Pediatric HSAT in this real-world population with mostly mild SDB symptoms demonstrates no significant first-night effect; however, moderate night-to-night variability exists in some children. Clinicians must consider this variability when interpreting HSAT results in cases where clinical presentation and single-night HSAT outcomes conflict; a multi-night assessment may be warranted to improve diagnostic accuracy.

**Supplementary Information:**

The online version contains supplementary material available at https://doi.org/10.1007/s11325-026-03677-0.

## Introduction

### Sleep-disordered breathing in children

Obstructive sleep-disordered breathing (SDB) encompasses a spectrum of breathing disorders characterized by recurrent partial or complete upper airway obstruction during sleep. This spectrum ranges from habitual snoring to obstructive sleep apnea (OSA). An extensive general population sample estimates the prevalence of primary snoring as 16%, mild SDB as 25%, and moderate SDB as 1.2% among children [1]. Polysomnography, an overnight study measuring various physiological parameters, including respiration, heart rate, and oxygen saturation, remains the gold standard for diagnosing pediatric SDB. Clinical decisions, such as adenotonsillectomy, often rely on the outcome of a single-night polysomnographic examination [2].

### Night-to-night variability and first night effect

The accuracy of sleep studies is influenced by several technical factors, including equipment type, signal quality, sensor placement, scoring, and interpretation, as well as environmental conditions during the test night and patient-related factors. While it is possible to control for all other variables, two physiological patient-related phenomena are of particular interest: night-to-night variability and the first-night effect. Night-to-night variability refers to random fluctuations in sleep parameters of an individual patient between consecutive nights, which can significantly affect diagnostic outcomes. In contrast, the first-night effect describes systematic differences observed between the initial night of testing and subsequent nights, often attributed to patient adaptation or environmental factors [3, 4]. These two phenomena can exist independently since night-to-night variability might not result in biased results from the first to subsequent nights, as observed in the first-night effect. Conversely, the lack of a first-night effect in the whole cohort does not exclude the night-to-night variability in the individual patients.

Extensive research in the adult population has established substantial night-to-night variability in the assessment of SDB [3–7]. However, pediatric studies investigating both night-to-night variability and the first-night effect have yielded conflicting results [8–15]. Some pediatric investigations reported first-night effects and notable variability [8–12], while others demonstrated neither systematic differences nor variability [13–15].

The first-night effect is often attributed to environmental unfamiliarity or disturbances caused by monitoring equipment [3, 4, 16]. Home sleep apnea testing (HSAT), conducted in the patient’s regular sleeping environment with less invasive recording devices, has been suggested as a potential approach to mitigate this effect [3, 5, 16–18]. Traditionally, pediatric sleep assessments have relied on in-laboratory polysomnography. However, polygraphy-based HSAT is frequently used due to greater accessibility, reduced waiting times, lower costs, and enhanced convenience for patients and caregivers [18–20]. Despite this growing trend, existing research primarily evaluates the first-night effect and variability using in-laboratory polysomnography. Limited evidence exists regarding these phenomena in home-based settings.

## Study aim and hypothesis

Given the inherent challenges of pediatric sleep studies and the common reliance on single-night testing, it is critical to understand the reliability and accuracy of a single-night HSAT in children, particularly regarding the first-night effect and night-to-night variability. This study aims to quantify both phenomena in a real-world population with relatively mild symptoms, specifically for pediatric HSAT, using respiratory polygraphy. The authors hypothesize that a first-night effect will be minimal or absent due to the reduced invasiveness and familiar setting of HSAT. However, we anticipate that night-to-night variability will be comparable to that previously reported for in-laboratory polysomnography.

## Materials and methods

### Study design

We conducted a retrospective analysis of pediatric HSAT utilizing respiratory polygraphy for children with suspected SDB. The sleep studies were performed during two consecutive nights between July 2023 and October 2024. In our institution, routine respiratory polygraphy is conducted routinely over two nights if scheduled on weekends. Existing recordings were retrospectively analyzed to evaluate the first-night effect and night-to-night variability. The study protocol received approval from the local ethics committee (Kantonale Ethikkommission Bern, project ID 2024 − 00870) and was conducted according to the Declaration of Helsinki [21]. Written consent was obtained from all caretakers.

## Patient selection and data collection

We included children aged two years or older with clinically suspected SDB who underwent HSAT using respiratory polygraphy over two consecutive nights. Due to the frequent occurrence of suboptimal signal quality, our institution performs respiratory polygraphy over two consecutive nights when testing is scheduled on weekends to mitigate data loss and improve diagnostic reliability. Patients younger than two years or with lacking consent were excluded. At our institution, patients with suspected non-obstructive sleep-related breathing disorders, such as central sleep apnea secondary to neurological diseases, do not undergo routine HSAT with respiratory polygraphy, as we believe these patients are better served with in-lab polysomnography. Therefore, these patients are not represented in our cohort. Otherwise, there were no exclusions based on comorbidities to preserve generalizability. Eligible recordings required acceptable technical quality, defined as a minimum of four hours of signal acquisition across all channels per night. Demographic and clinical information, including age, sex, weight, height, and relevant comorbidities, was extracted from electronic medical records. Asthma was diagnosed in accordance with the Global Initiative for Asthma (GINA) guidelines [22], based on the presence of respiratory symptoms (wheeze, shortness of breath, chest tightness, cough) together with objective evidence of variable expiratory airflow limitation on spirometry (e.g. positive bronchodilator reversibility test and/or positive bronchial provocation test). Allergic rhinitis was diagnosed according to the Allergic Rhinitis and its Impact on Asthma (ARIA), based on typical nasal symptoms (itching, sneezing, nasal obstruction) in combination with documented allergic sensitization demonstrated by skin prick testing and/or serum-specific immunoglobulin E [23].

### Home sleep apnea testing and analysis of signals

HSAT was performed using respiratory polygraphy (SOMNOtouch™ RESP, SOMNOmedics AG, Randersacker, Germany). All recordings were performed in the patient’s home and familiar sleeping environment. Caretakers were instructed to provide optimal conditions to have accurate and representative HSAT results. According to the American Academy of Sleep Medicine guidelines, all recordings were manually reviewed and scored by experienced sleep specialists [24]. Sleep time was determined by manually reviewing the signal channels and marking sleep onset and awakening. Recordings with less than 4 h of sleep time of good signal quality were excluded.

### Statistical analysis

The primary endpoint for assessing the first-night effect was the difference in the apnea-hypopnea index (AHI) between the two consecutive nights. Secondary endpoints included differences between nights in oxygen desaturation index (ODI), total sleep time, mean oxygen saturation, percentage of time with SpO2 below 90%, respiration rate, and heart rate. The first-night effect was evaluated as a systematic difference between the two nights, and significance testing was performed with the Wilcoxon signed-rank test. Variability, defined as the absolute difference between the two nights, was analyzed for associations with comorbidities, such as allergies and asthma, using the Kruskal-Wallis test. Linear regression models were applied to assess the influence of age, tonsil size, and adenoid size on differences between nights. Agreement and variability between the two measurements were visualized using Bland-Altman plots [25], violin plots, and box plots. To describe the clinical relevance of night-to-night variability, tables were created to represent the presence and severity of OSA in the participants for each night. Misclassification of diagnosis was calculated using an AHI threshold of 1/h, and misclassification of disease severity was calculated using an OSA severity classification of mild (AHI 1–5/h), moderate (AHI 5–10/h), and severe (AHI > 10/h) [2]. Raw agreement and Cohen’s kappa were calculated to assess diagnostic consistency between nights. Cohen’s kappa reliability was interpreted according to Landis and Koch as follows: poor (< 0.20), fair (0.21–0.40), moderate (0.41–0.60), good (0.61–0.80), and excellent (> 0.80) [26]. All statistical analyses were conducted using R software (version 4.5.1, R Foundation for Statistical Computing, Vienna, Austria).

## Results

### Patient flow and cohort description

Between July 2023 and October 2024, 118 pediatric patients underwent HSAT over two consecutive nights. Of these, seven children were excluded as they were under two years of age, one due to a lack of consent for data use, and 60 patients were excluded due to insufficient data quality on one or both nights. The final cohort consisted of 48 patients with a median age of 6.7 years (interquartile range, IQR: 4.3–8.7 years), of whom 36 (75%) were male. The median height percentile was 46 (IQR: 23–65), and the median weight percentile was 49 (IQR: 24–79). Allergies were present in 16 patients (33%), and asthma was confirmed in four patients (8%) and suspected in an additional three patients (6%). Prior treatments included intranasal corticosteroids in 27 children (56%) and adenotonsillectomy in 11 children (23%). Comorbidities were identified in 19 children and categorized into pulmonary disease (*n* = 5, 10%), syndrome-like disorder (*n* = 4, 8%), and other comorbidities (*n* = 10, 21%).

### First-night effect

The first-night effect refers to a systematic difference between the first night and subsequent nights. In our study, we assessed the differences in sleep parameters of the entire cohort between the two consecutive nights, as detailed in Table 1. In our cohort, no significant systematic differences were observed for all measured sleep parameters between the first and second night, including sleep time, respiratory parameters, oxygen saturation, and heart rate.

**Table 1:** Comparison of sleep parameters between the first and second night

Variable	Night 1	Night 2	Median Difference	*p* value
Sleep Time (hours)	7.5 [ 6.7, 8.2]	7.5 [ 6.9, 8.2]	0.0 [-0.3, 0.4]	0.86
Apnea-hypopnea index (events per hour)	1.0 [ 0.6, 2.0]	1.0 [ 0.7, 1.9]	0.1 [-0.2, 0.4]	0.64
Obstructive apnea-hypopnea index (events per hour)	0.6 [ 0.3, 0.9]	0.5 [ 0.3, 1.0]	0.0 [-0.2, 0.2]	0.93
Apnea index (events per hour)	0.9 [ 0.5, 1.4]	0.8 [ 0.4, 1.3]	0.1 [-0.1, 0.3]	0.32
Central apnea index (events per hour)	0.4 [ 0.2, 0.9]	0.4 [ 0.2, 0.9]	0.1 [-0.1, 0.3]	0.54
Hypopnea index (events per hour)	0.2 [ 0.0, 0.5]	0.1 [ 0.0, 0.5]	0.1 [-0.1, 0.2]	0.46
Respiratory disturbance index (events per hour)	1.0 [ 0.6, 1.9]	1.1 [ 0.7, 1.9]	0.1 [-0.2, 0.3]	0.67
Mean Apnea (seconds)	9.1 [ 7.8, 10.9]	9.9 [ 8.4, 11.7]	-0.6 [-1.6, 0.4]	0.25
Maximum Apnea (seconds)	13.0 [11.0, 17.0]	14.0 [10.0, 16.2]	0.0 [-2.5, 2.5]	0.88
Supine time (% of sleep time)	53.0 [39.0, 64.2]	51.9 [33.9, 61.7]	3.0 [-3.1, 9.6]	0.31
Supine apnea-hypopnea index (events per hour)	1.3 [ 0.6, 2.2]	1.0 [ 0.6, 2.2]	0.2 [-0.3, 0.7]	0.43
Supine apnea index (events per hour)	0.9 [ 0.3, 1.8]	0.8 [ 0.4, 1.6]	0.2 [-0.2, 0.6]	0.16
Supine oxygen desaturation index (events per hour)	2.5 [ 1.0, 5.0]	1.9 [ 0.9, 4.9]	0.4 [-0.6, 1.2]	0.29
Oxygen desaturation index (events per hour)	2.6 [ 1.2, 3.9]	2.3 [ 1.2, 4.2]	0.1 [-0.7, 0.9]	0.74
Mean oxygen saturation (%)	98.0 [97.0, 99.0]	98.0 [97.0, 99.0]	0.5 [ 0.0, 1.0]	0.17
Time below 90% oxygen saturation (% of sleep time)	0.0 [ 0.0, 0.0]	0.0 [ 0.0, 0.1]	-0.1 [-6.2, 0.1]	0.14
Respiration rate (breaths per minute)	17.3 [16.1, 19.0]	17.3 [16.3, 20.0]	-0.5 [-1.2, 0.3]	0.18
Mean heart rate (beats per minute)	79.0 [73.8, 86.2]	80.0 [74.0, 86.0]	0.0 [-1.5, 1.5]	0.97

Data are presented as median (interquartile range, IQR). Statistical differences between nights were assessed using the Wilcoxon signed-rank test

### Night-to-night Variability and accuracy of OSA diagnosis and severity

**Fig. 1:**
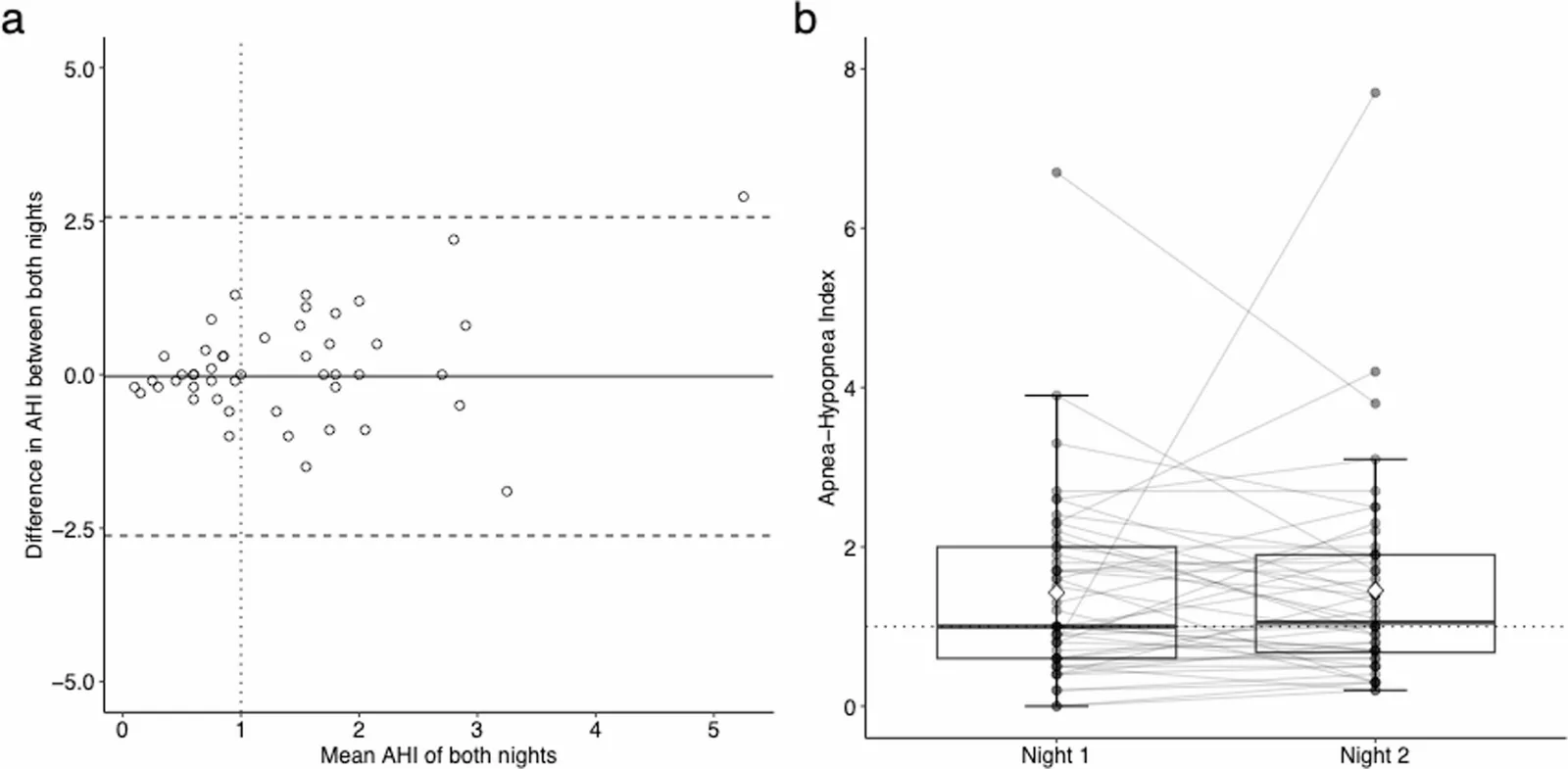
Night-to-night Variability of the Apnea-Hypopnea Index (AHI). (**a**) Bland-Altman plot demonstrating the differences in AHI between two nights, including the mean difference (solid line) and limits of agreement (± 1.96 standard deviations, dashed lines). (**b**) Boxplots illustrating the distribution of AHI values per night. A diamond symbol indicates the mean, and the dotted horizontal line marks the diagnostic threshold for sleep apnea (AHI = 1 event/hour)

**Fig. 2:**
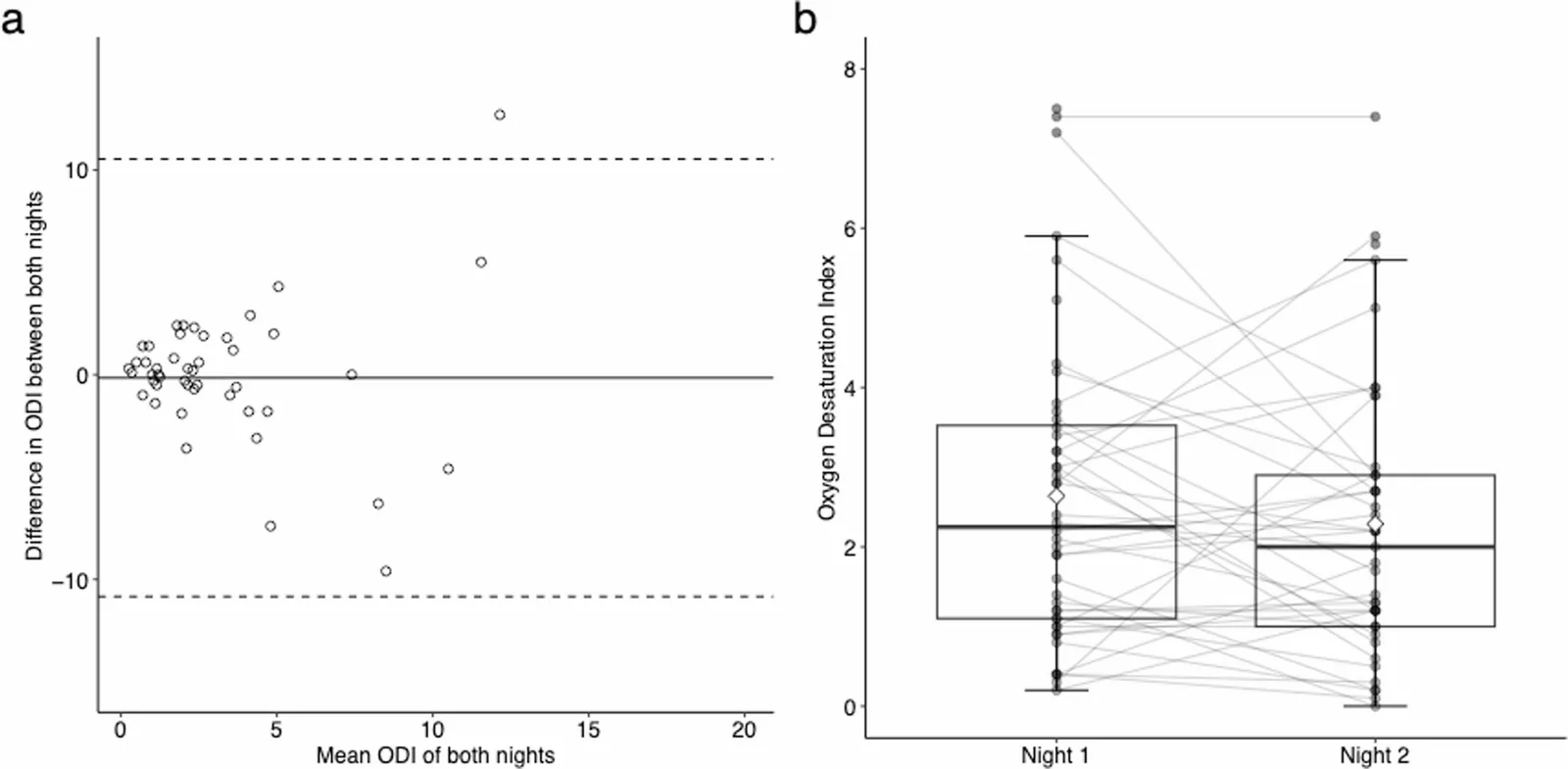
Night-to-night variability of the Oxygen Desaturation Index (ODI). (**a**) Bland-Altman plot depicting the differences in ODI values between two consecutive nights, with the mean difference (solid line) and limits of agreement (± 1.96 standard deviations, dashed lines). (**b**) Boxplots showing the ODI distribution per night, with the mean value represented by a diamond symbol

**Fig. 3:**
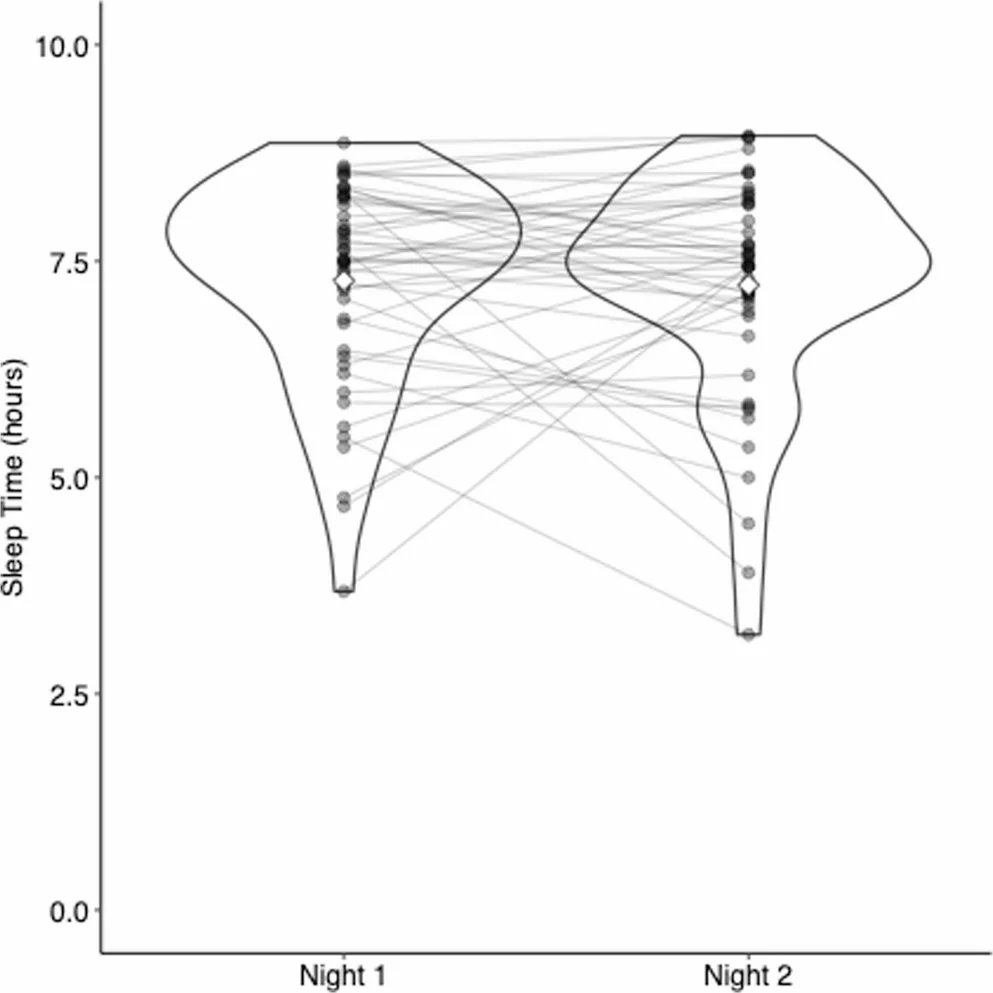
Night-to-Night Variability of Total Sleep Time. Violin plot illustrating the distribution and variability of total sleep time (hours) between two consecutive nights. Diamond symbols represent mean values for each night

**Fig. 4:**
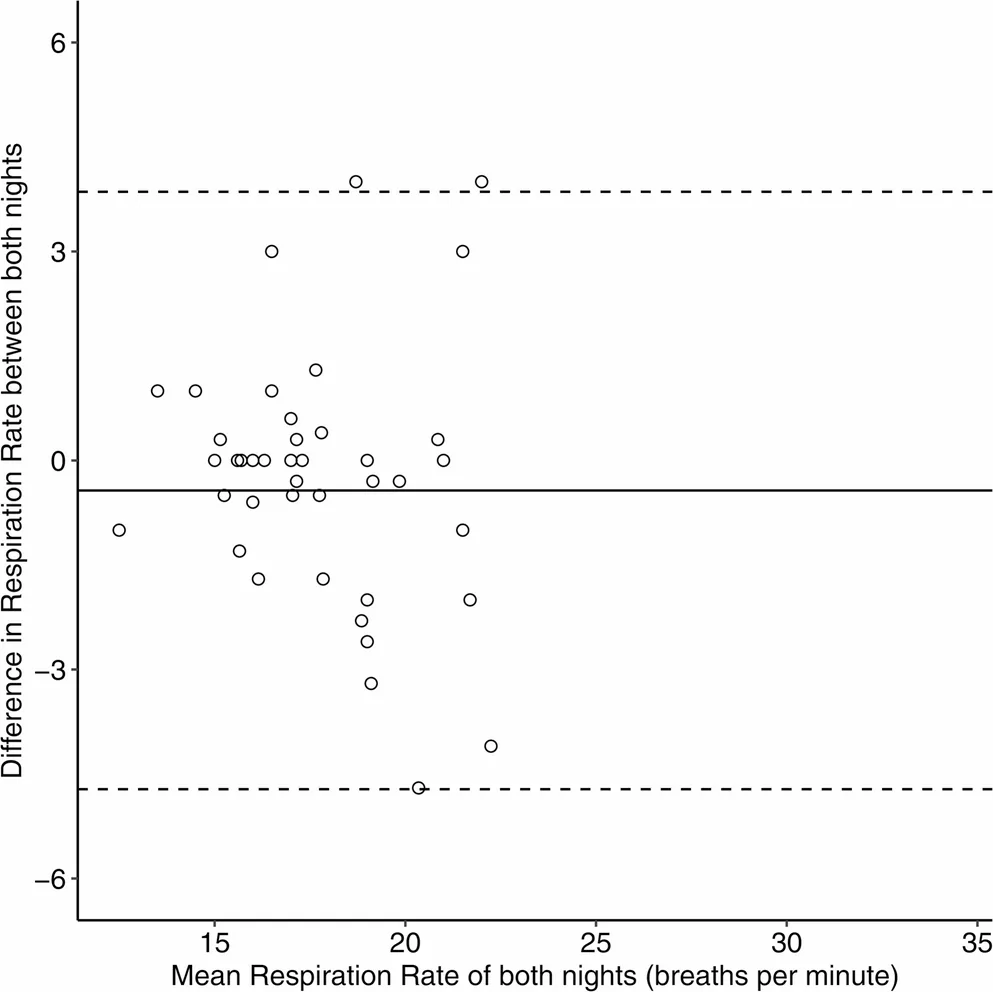
Night-to-night Variability of RespiRation rate. Bland-Altman plot comparing respiration rates (breaths per minute) between two consecutive nights, including the mean difference (solid line) and limits of agreement (± 1.96 standard deviations, dashed lines)

**Fig. 5:**
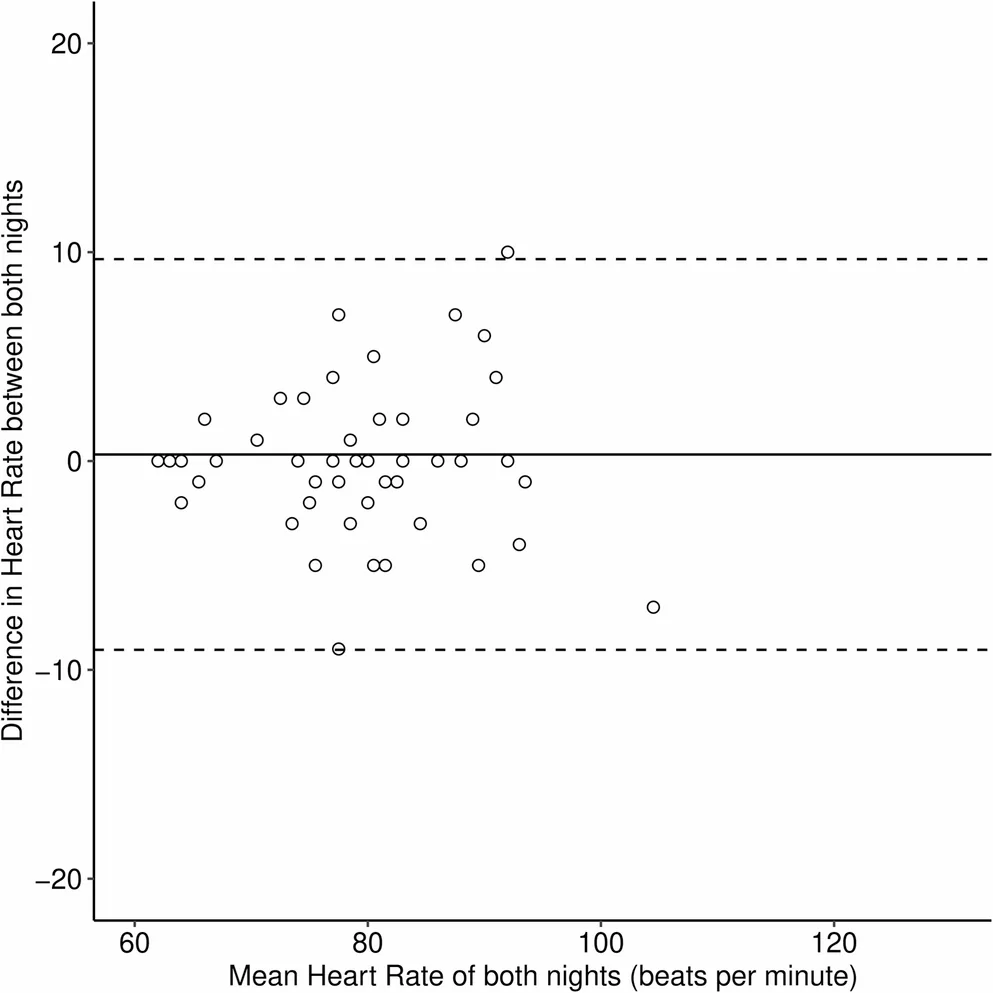
Night-to-night variability of Heart Rate. Bland-Altman plot illustrating differences in heart rate (beats per minute) between consecutive two nights, showing the mean difference (solid line) and limits of agreement (± 1.96 standard deviations, dashed lines)

When examining individual night-to-night variability, outcomes varied significantly for some children between nights. Figure 1 illustrates the variability in AHI between nights. A similar variability pattern was noted for the ODI, as shown in Fig. 2, further corroborating these findings. Additional parameters, including total sleep time (Fig. 3, Supplementary Information Fig. 1), respiration rate (Fig. 4), and heart rate (Fig. 5), also exhibited considerable night-to-night variability in some individuals, without a systematic bias between nights. This led to a misclassification of OSA severity and misdiagnosis in some patients. OSA was diagnosed in 23 out of 48 patients (48%) on the first night and 24 out of 48 patients (50%) on the second night. Diagnostic consistency for OSA between nights showed a raw agreement of 77%, corresponding to a moderate agreement (Cohen’s kappa = 0.54). Similarly, the agreement for categorizing OSA severity was moderate (Cohen’s Kappa = 0.46), with severity misclassification observed in 14 out of 48 patients (29%) when comparing the results from two consecutive nights. We observed misclassification primarily between no and mild OSA. In one patient, the OSA classification changed from no to moderate OSA (Table 2). When assuming the mean of both nights to be the accurate OSA diagnosis, the sensitivity was 0.90, and the specificity was 0.82. The positive predictive value was 0.86, and the negative predictive value was 0.88.

**Table 2:** Agreement in obstructive sleep apnea severity between the first and second night

		Night 1			
		No	Mild	Moderate	Severe
Night 2	No	**14**	6	0	0
	Mild	6	**20**	1	0
	Moderate	1	0	**0**	0
	Severe	0	0	0	**0**

Data are presented as the number of patients within each severity category for both nights

### Subgroup analysis

We investigated age as a possible influencing factor on the night-to-night variability (Supplementary Information Fig. 2). Linear regression analysis revealed no association between age and variability in AHI (β = -0.04, 95% CI: -0.16 to 0.08, *p* = 0.54). Although visual assessment suggested a possible decrease in absolute variability with increasing age, this trend was not statistically significant (β = -0.08, 95% CI: -0.18 to 0.02, *p* = 0.11).

Comorbidities were categorized into pulmonary disease, syndrome-like disorders, and other conditions for further analysis (Supplementary Information Table 1). No significant differences in the magnitude of night-to-night variability were found between the comorbidity groups. For all collected sleep parameters, the magnitude of night-to-night variability was not influenced by tonsil size, adenoid size, or asthma. The variability of the respiration rate was significantly lower in children with allergies compared to those without (median (interquartile range) 0.30 (0.53) vs. 1.70 (2.45, *p* < 0.01), while otherwise no effect of allergies on variability was found.

## Discussion

### Overview

This study assessed night-to-night variability and the first-night effect in HSAT using respiratory polygraphy over two consecutive nights in children with suspected SDB and rather mild to moderate symptoms. Our findings indicate no significant first-night effect for all tested parameters, including sleep time, respiratory parameters, oxygen saturation, and heart rate. However, we observed night-to-night variability in respiratory and oxygenation parameters in some children, which resulted in the misclassification of OSA diagnosis and severity in almost one-third of the children. When analyzing influencing factors on the variability, only allergies seemed to reduce the variability of respiration rate. Otherwise, no predictors of variability could be identified. Consequently, single-night measurements can lead to inaccuracies in diagnosing OSA and assessing its severity, even in this population with only mild disease severity. The degree of variability and misclassification observed is consistent with previous studies in adult patients [3] and children [11].

### Comparison to previous studies

While we identified considerable variability, no systematic differences emerged between the first and second nights. Previous studies attribute the first-night effect predominantly to disturbances caused by unfamiliar sleep environments or recording equipment [3–5, 16]. HSAT typically shows reduced or absent first-night effects compared to laboratory settings [3, 13]. The familiar home environment appears particularly beneficial for children, potentially mitigating the first-night effect commonly observed in in-laboratory studies and adults [4, 10]. Alternatively, children may either remain undisturbed by recording devices or fail to habituate within the two nights.

A meta-analysis by Ding et al., which analyzed polysomnography data, reported first-night effects for sleep onset latency, sleep efficiency, and certain sleep stages, but not for the AHI [27]. Since our study utilized respiratory polygraphy, we were unable to evaluate sleep architecture changes, which may be more susceptible to variability than respiratory events. This meta-analysis also found that the first-night effect is less pronounced in younger children, which supports the idea of fewer disturbances or adaptation in younger patients [27].

### Implications for the clinic

Our results underline the necessity for cautious interpretation of single-night HSAT due to inherent night-to-night variability. Interpretation of HSAT findings that are inconsistent with the clinical presentation or reported symptoms is challenging. Night-to-night variability may account for discrepancies between clinical presentation and objective sleep apnea testing, and clinicians should consider multi-night testing in these cases. These findings should therefore be regarded as complementary diagnostic information that should be interpreted by the treating clinician in the context of symptoms, history, and overall presentation. Similarly, treatment evaluations should consider variability, as it may mask genuine therapeutic effects. Since conventional sleep apnea testing is resource-intensive, several studies have investigated the use of wearables [28–31]. Due to their small form factor, they would be instrumental in pediatric sleep testing over multiple nights. However, wearables available to consumers are often not validated, especially in children, making their interpretability challenging [31–33]. 

### Strengths and limitations

The strengths of this study include the relatively large and clinically unselected, representative cohort of children with suspected SDB. Although the majority of children have only mild to moderate disease, this is representative of the population of a tertiary hospital that is assessed via the HSAT. Children who have clinically suspected comorbid sleep disorders, such as neurological diseases, that are associated with severe OSA are usually scheduled for polysomnography testing within the hospital. This may partially explain the overrepresentation of mild and moderate SDB, although extensive studies find comparable proportions [1]. Furthermore, evaluating night-to-night variability using HSAT in a pediatric population addresses an understudied field of research with limited existing literature. In the authors’ opinion, evaluating the accuracy of pediatric HSAT is essential, as HSAT is widely used to avoid long waiting times and the cost of in-laboratory polysomnography.

There are limitations: In our cohort, we diagnosed mainly mild SDB. While relative variability between nights is considerable, the absolute difference is minor. Since the ground truth cannot be known, we used the mean of both measurements as the correct classification. However, this may overestimate the accuracy, since the mean correlates more with the individual measurements than the hypothetical ground truth. Further, negative and positive predictive values are dependent on the prevalence in the cohort. Caretakers were instructed to ensure optimal conditions for accurate and representative sleep apnea testing. However, unobserved external factors may still have influenced the results and contributed to variability. Due to the retrospective study design, several factors related to sleep could not be investigated, such as ethnic and socioeconomic status [34–36]. Future prospective studies should investigate these influencing factors on sleep. Furthermore, we assessed only two consecutive nights, preventing the evaluation of long-term variability. For an unbiased sample, we consecutively recruited all patients from July 2023 to October 2024. Most children in the cohort presented with mild or no OSA, potentially limiting generalizability to more severely affected children. A considerable proportion of the initial cohort’s recordings had to be excluded due to insufficient data quality. Furthermore, sleep position data in pediatric recordings often lack accuracy, and HSAT did not record electroencephalogram and limb movements, precluding an analysis of positional effects on variability. Lastly, respiratory polygraphy does not allow for the assessment of sleep stages.

### Recommendations for Future Research

Future research should explore night-to-night variability over extended periods and in larger pediatric cohorts, potentially improving the understanding of influencing factors. However, long-term monitoring in pediatric populations remains challenging due to device discomfort and size limitations. There is a clear need for smaller, validated, minimally disruptive sleep monitoring devices suitable for pediatric use. Since few wearables are validated, especially for children, future research should investigate these devices [31–33]. Long-term measurements using wearables could also be used to analyze other influencing factors on pediatric sleep, such as socioeconomic status [34–37]. In-lab and home sleep testing should also be directly compared in terms of night-to-night variability and the first-night effect. Additionally, future studies should examine relationships between objective sleep measurements and subjective clinical assessments, including caregiver-reported disease burden.

## Conclusion

Our findings indicate no systematic first-night effect in pediatric HSAT using respiratory polygraphy. However, moderate night-to-night variability in respiratory and oxygenation parameters underscores the risk of diagnostic inaccuracies based on single-night testing. Clinicians should interpret HSAT results cautiously and in conjunction with overall clinical presentation and symptoms, especially if they differ from clinical presentation. Interpretation of clinically discordant HSAT results is challenging. Therefore, multi-night assessments should be considered to ensure accurate diagnosis and correct treatment selection.

## Supplementary Information

Below is the link to the electronic supplementary material.

Supplementary Material 1

## Data Availability

The data supporting the findings of this study are available on request from the corresponding author, Samuel Tschopp.
