# Supplementary material for: Night-to-night variability in respiratory outcomes from home sleep testing in children with suspected sleep-disordered breathing: a retrospective analysis using real-world data
Source: Sleep Breath. 2026 May 16;30(3):163. doi: 10.1007/s11325-026-03677-0 (PMC13652924; doi:10.1007/s11325-026-03677-0)
Supplement: Supplementary file 1 [file 11325_2026_3677_MOESM1_ESM.docx]

**Supplementary Information**

**Supplementary Information Fig. 1 Night-to-Night Variability of the Sleep Time.** Bland-Altman plot depicting the differences in sleep time between two consecutive nights, with the mean difference (solid line) and limits of agreement (±1.96 standard deviations, dashed lines).

**Supplementary Information Fig. 2 Apnea-Hypopnea Index Variability between Nights by Age (years).** The Bland-Altman plot shows the mean difference (solid line) and limits of agreement (±1.96 standard deviations, dashed lines).

**Supplementary Information Table 1 Influence of Comorbidities on Night-to-Night Variability.** Absolute differences between the two nights are presented as median and interquartile range, and statistical significance is assessed using the Kruskal-Wallis rank sum test.

| **Variable** | **None** | **Syndrome-like Disorder** | **Pulmonary Disease** | **Other** | **P value** |
| --- | --- | --- | --- | --- | --- |
| n | 29 | 4 | 5 | 10 |  |
| Sleep Time (hours) | 0.6 [0.2, 0.8] | 0.5 [0.0, 1.1] | 1.5 [0.2, 2.3] | 1.2 [0.7, 2.2] | 0.13 |
| Apnea-hypopnea index (events per hour) | 0.3 [0.1, 0.8] | 0.4 [sz0.0, 1.1] | 0.9 [0.6, 1.0] | 0.5 [0.3, 1.0] | 0.41 |
| Obstructive apnea-hypopnea index (events per hour) | 0.2 [0.1, 0.3] | 0.4 [0.2, 1.1] | 0.5 [0.2, 0.6] | 0.3 [0.2, 0.5] | 0.18 |
| Apnea index (events per hour) | 0.3 [0.1, 0.7] | 0.6 [0.3, 1.0] | 0.5 [0.2, 0.6] | 0.5 [0.1, 0.9] | 0.79 |
| Central apnea index (events per hour) | 0.2 [0.1, 0.5] | 0.2 [0.0, 0.6] | 0.2 [0.1, 0.5] | 0.1 [0.0, 0.5] | 0.90 |
| Hypopnea index (events per hour) | 0.1 [0.0, 0.3] | 0.2 [0.1, 1.0] | 0.5 [0.1, 0.7] | 0.0 [0.0, 0.2] | 0.26 |
| Respiratory disturbance index (events per hour) | 0.3 [0.1, 0.8] | 0.5 [0.2, 1.1] | 0.9 [0.6, 1.0] | 0.5 [0.3, 1.0] | 0.43 |
| Mean Apnea (seconds) | 2.1 [0.6, 3.8] | 3.9 [0.4, 8.1] | 1.6 [0.3, 3.2] | 1.6 [0.9, 2.0] | 0.71 |
| Maximum Apnea (seconds) | 4.0 [1.0, 8.0] | 11.0 [7.5, 12.8] | 2.0 [1.0, 2.0] | 2.0 [0.2, 4.5] | 0.37 |
| Supine time (% of sleep time) | 0.6 [0.3, 1.2] | 0.3 [0.0, 0.6] | 0.4 [0.1, 0.5] | 0.5 [0.1, 1.1] | 0.44 |
| Oxygen desaturation index (events per hour) | 1.4 [0.6, 2.4] | 3.0 [0.5, 10.3] | 1.9 [0.6, 7.4] | 0.7 [0.4, 2.2] | 0.72 |
| Mean oxygen saturation (%) | 0.0 [0.0, 1.0] | 0.5 [0.0, 2.2] | 1.0 [1.0, 1.0] | 0.0 [0.0, 1.0] | 0.52 |
| Time below 90% oxygen saturation (% of sleep time) | 0.0 [0.0, 0.1] | 0.0 [0.0, 11.1] | 0.0 [0.0, 0.0] | 0.0 [0.0, 0.1] | 0.72 |
| Respiration rate (breaths per minute) | 0.4 [0.0, 1.0] | 1.3 [0.2, 2.8] | 2.6 [0.3, 4.7] | 1.7 [0.6, 3.1] | 0.11 |
| Mean heart rate (beats per minute) | 2.0 [0.0, 3.0] | 4.5 [3.0, 5.0] | 4.0 [2.0, 9.0] | 2.0 [0.2, 4.2] | 0.32 |
